# Metal–Organic Framework Optical Thermometer Based on Cr^3+^ Ion Luminescence

**DOI:** 10.1021/acsami.2c19957

**Published:** 2023-01-30

**Authors:** Adam Kabański, Maciej Ptak, Dagmara Stefańska

**Affiliations:** Institute of Low Temperature and Structure Research, Polish Academy of Sciences, Wrocław50-422, Poland

**Keywords:** hybrid perovskite, luminescence, thermometry, chromium(III) ions, temperature sensing, noncontact optical thermometer

## Abstract

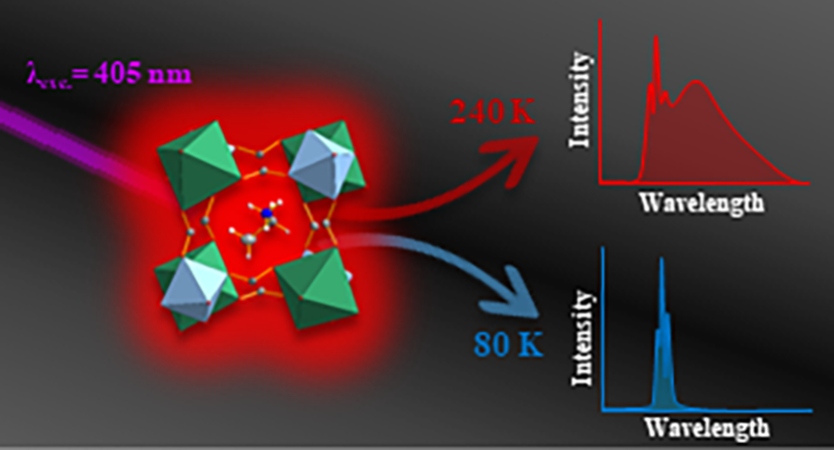

Metal–organic frameworks with perovskite structures have recently attracted increasing attention due to their structural, optical, and phonon properties. Herein, we report the structural and luminescence studies of a series of six heterometallic perovskite-type metal–organic frameworks with the general formula [EA]_2_NaCr_*x*_Al_1–*x*_(HCOO)_6_, where *x* = 1, 0.78, 0.57, 0.30, 0.21, and 0. The diffuse reflectance spectral analysis provided valuable information, particularly on crystal field strength (*D*q/*B*) and energy band gap (*E*_g_). We showed that the *D*q/*B* varies in the 2.33–2.76 range depending on the composition of the sample. Performed Raman, XRD, and lifetime decay analyses provided information on the relationship between those parameters and the chemical composition. We also performed the temperature-dependent luminescence studies within the 80–400 K range, which was the first attempt to use an organic–inorganic framework luminescence thermometer based solely on the luminescence of Cr^3+^ ions. The results showed a strong correlation between the surrounding temperature, composition, and spectroscopic properties, allowing one to design a temperature sensing model. The temperature-dependent luminescence of the Cr^3+^ ions makes the investigated materials promising candidates for noncontact thermometers.

## Introduction

Over the past few years, perovskite materials with the general formula ABX_3_ have become a significant object of study. One of the most noteworthy groups of these materials is hybrid compounds containing organic cations A (e.g., ammonium and methylammonium), divalent metal ions B (e.g., Pb^2+^ and Sn^2+^), and halide ligands X (e.g., I^–^ and Cl^–^).^1,2^ Hybrid compounds have been particularly useful in thin-film photovoltaic devices.^3,4^ Due to their specific properties, such as ferroelectricity,^5−7^ colossal magnetoresistance,^8,9^ magnetocaloric effect,^10,11^ and unique optical properties,^1,12,13^ they can be implemented in various applications. The characteristics of the perovskite-like materials can be significantly tuned by replacing the A and B ions and X linkers.^14−16^

Formate-based metal–organic frameworks (MOFs) with perovskite structures have attracted a lot of attention due to their various properties, such as multiferroicity, ferroelectricity, luminescence, and magnetic effects.^17−21^ The bimetallic compounds with the general formula [A]_2_M^I^M^III^(HCOO)_6_, where A = EA^+^ (ethylammonium), DMA^+^ (dimethylammonium), M^I^ = Na^+^ and K^+^, and M^III^ = Cr^3+^, Al^3+^, and Fe^3+^, exhibit unique, especially temperature-induced properties.^14,16,19^ The origin of this phenomenon is related to order–disorder phase transitions and changes in energy level populations caused by the change in temperature.

Bimetallic perovskite-like MOFs also exhibit interesting luminescence properties.^14,22−24^ Particularly, the subgroup of chromium-based phosphors is noteworthy due to its strong temperature sensitivity and weak concentration quenching.^22,23^ The heterometallic MOFs containing chromium ions exhibit temperature-dependent luminescence properties and may be used for noncontact temperature detection.^14^

The spectroscopic properties of the transition metal (TM) ions, such as Cr^3+^, are strongly dependent on the local environment, particularly the type of crystal field and temperature.^14,25−27^ Trivalent chromium ions exhibit two main emission bands: narrow spin-forbidden ^2^E_g_ → ^4^A_2g_ transitions around 700 nm and broad spin-allowed ^4^T_2g_ → ^4^A_2g_ transitions near 750 nm. The narrow emission occurs in a strong crystal field, whereas broad emission takes place when the material exhibits a weak crystal field.^28,29^ The materials with intermediate crystal field strength exhibit both types of emissions. Furthermore, the change of temperature strongly affects the intensities of bands, which was reported as a potentially useful feature for temperature sensing.^27^ The concentration of the Cr^3+^ ions affects not only the intensity of emission but also may influence the dominant emission band due to the change of the crystal structure.^26^

Luminescence noncontact thermometry is a novel scientific approach for temperature measurements that has attracted a lot of attention.^30−36^ Noncontact temperature sensing has a high potential for application in industrial, scientific, biomedical, and technological fields due to a variety of advantages, such as micro- and nanosized implementation possibility, high accuracy, and fast response. The general measurement mechanism is mainly based on thermally induced changes in the quality of luminescence spectra, such as peak intensities, positions, or decay lifetimes.^31,37^ The most promising approach is based on the examination of the parameters known as fluorescence intensity ratio (FIR or Δ), which is calculated by comparing intensities of two emission peaks.^27^ FIR-based methods are particularly useful due to the minimalization of an influence of measurement conditions.^27,36^

The temperature calculation can be based on the emissions originating from individual dopants or codopants incorporated into the structure of the material. Luminescent materials used for temperature sensing can be divided into several groups according to their size (nano- and microthermometers), change of the wavelength (down- and up-converting materials), and number of emission centers (single and dual emission centers). Another subgroup of thermometric materials is compounds containing rare-earth (RE) metal ions.^27,38^

The application of host materials exhibiting luminescence properties enables the analysis based on the emission peaks of both host and dopant. The vast majority of research involves inorganic host materials containing RE metal ions as dopants.^13,39−41^ Thermal sensing solutions based on transition metal ions are not so popular; however, promising results have been reported for double perovskites codoped with vanadium ions.^13^

Another noteworthy approach is the incorporation of trivalent chromium ions, which can be a valuable direction for RE-free luminescence thermometer design.^42−44^ The potential of implementation of Cr^3+^ ions for high thermal sensing has been reported for several inorganic materials.^13,45,46^ However, up to date, such solutions based on MOFs have not been proposed.^13,43,44^ Another promising approach is using mixed systems containing chromium ions as well as lanthanide ions.^30,47^

Herein, we report the preparation and structural and optical characteristics of the first MOF-type luminescence thermometers based solely on spectroscopic properties of the Cr^3+^ ions, i.e., [EA]_2_NaCr_*x*_Al_1–*x*_(HCOO)_6_, where *x* = 1, 0.78, 0.57, 0.30, 0.21, and 0. The investigated materials exhibit significant temperature-dependent emission, simultaneously related to chromium ion concentration. In this work, we attempt to describe the effect of the composition of the material and the strength of the crystal field on the spectroscopic properties. Particular attention is given to the possibility of the implementation of investigated materials as noncontact luminescence thermometers. To achieve this purpose, we performed spectroscopic analysis in a broad temperature range.

## Experimental Section

### Materials and Instrumentation

All precursors (analytical grade) were commercially available and were used without further purification. The synthesis was performed on an Ertec Magnum II microwave reactor with a standard Teflon vessel. The powder X-ray diffraction (XRD) patterns were obtained on an X’Pert Pro X-ray diffraction system equipped with a PIXcel detector, a focusing mirror, and Soller slits for CuKα radiation (λ = 1.54056 Å). The Raman spectra were measured using a Bruker MultiRAM spectrometer with 2 cm^–1^ resolution. A 1064 nm wavelength YAG:Nd laser was used as an excitation source. The diffuse reflectance spectra were obtained using a Varian Cary 5E UV–VIS–NIR spectrometer. The temperature-dependent emission spectra were obtained with a Hamamatsu PMA-12 photonic multichannel analyzer combined with a BT-CCD sensor. As an excitation source, a 405 nm laser diode was used. The temperature was controlled by a Linkam THMS600 stage. For lifetime measurements, a Ti-sapphire laser pumped with Nd:YAG was used as the excitation source. To record decay profiles, the digital oscilloscope Tektronix MDO3052 was used. The compositions of samples were determined with energy-dispersive X-ray spectroscopy (EDS) measurement using a FEI NOVA NanoSEM 140 scanning electron microscope.

### Synthesis

A series of [EA]_2_NaCr_*x*_Al_1–*x*_(HCOO)_6_, where *x* = 1, 0.78, 0.57, 0.30, 0.21, and 0, were prepared using the microwave-assisted solvothermal method. Exemplarily, to grow [EA]_2_NaCr_0.78_Al_0.22_(HCOO)_6_ crystals, 3.2 mmol (0.8526 g) of CrCl_3_·6H_2_O, 0.8 mmol (0.3900 g) of Al(ClO_4_)_3_·9H_2_O, 4 mmol of EA·HCl (0.3262 g), and 8.8 mmol of HCOONa (0.5985 g) were dissolved in 15 mL of water. Afterward, 25 mL of *N*-ethylformamide and 5 mL of 98% HCOOH were added to the prepared solution. The mixture was subsequently transferred to a microwave reactor containing a Teflon vessel. The reaction was maintained at 140 °C for 16 h and then cooled to room temperature. The solution was kept undisturbed for 24 h. Next, obtained crystals were separated from the mother liquid and dried at 50 °C. The obtained crystals of the [EA]_2_NaAl(HCOO)_6_ sample were colorless. In contrast, crystals of materials containing Cr^3+^ ions were purple or dark purple depending on the chromium concentration. The exact quantities of precursors used for the preparation together with nominal and experimentally determined compositions of each sample are listed in Table S1. The performed EDS analysis provided information about the real concentration of Cr^3+^ ions, which is used in the further part of the paper.

## Results and Discussion

### Structural Properties

**Figure 1:**
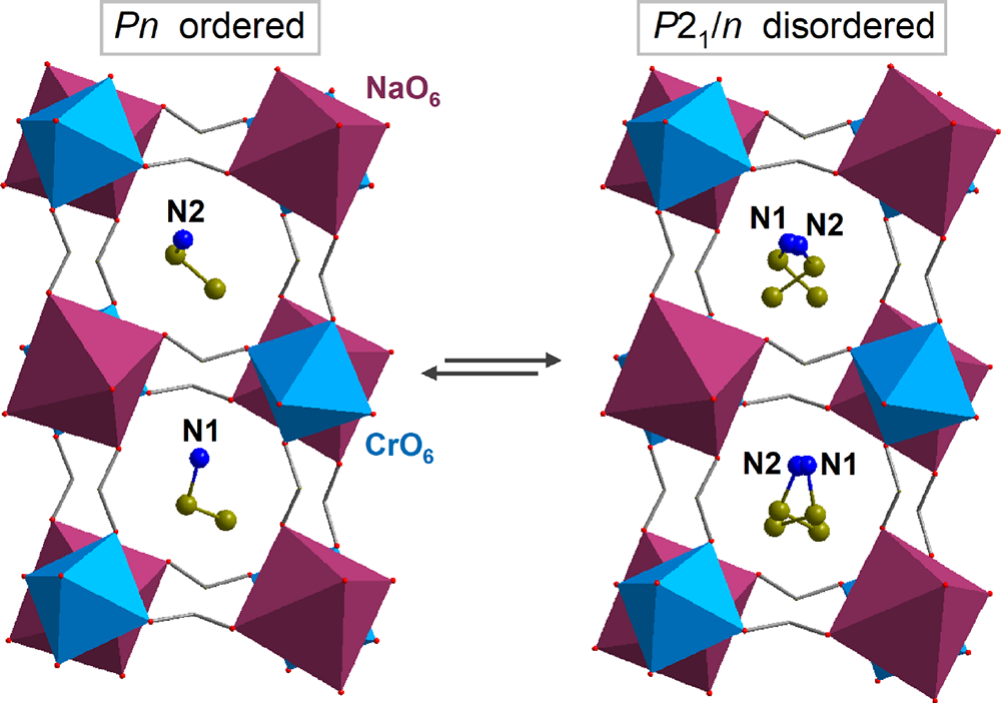
Crystal structure of EANaCr in the high-temperature *P*2_1_/*n* and low-temperature *Pn* phases. H atoms are omitted for clarity.

Both [EA]_2_NaCr(HCOO)_6_ (EANaCr) and [EA]_2_NaAl(HCOO)_6_ (EANaAl) crystallize in the monoclinic, polar space group *Pn*. Previously published results have shown that the space group transformation into a *P*2_1_/*n* space group occurs at around 373 K for EANaCr and 369 K for EtANaAl.^6^ The crystal structure in both phases exhibits a perovskite-like topology composed of alternatively distributed octahedral units of CrO_6_/AlO_6_ and NaO_6_. A 3D metal-formate framework comprises voids occupied by EA^+^ cations (see Figure 1). In the high-temperature *P*2_1_/*n* phase, organic cations are dynamically disordered over two independent positions that are occupied with roughly 50% probability. In the low-temperature *Pn* phase, due to the strengthening of hydrogen bonds, the metal-formate framework distorts, voids shrink, and the thermal motions of EA^+^ cations are suppressed. The arrangement of EA^+^ dipole moments in the ordered *Pn* phase is responsible for the polar properties of EANaCr and EANaAl.^6^

The cell volume in the [EA]_2_NaCr_*x*_Al_1–*x*_(HCOO)_6_ series is dependent on the type of metal ion; the decrease in volume was reported, while the Cr^3+^ ions were replaced with Al^3+^ ions. Such a phenomenon is related to the different ionic radii of Cr^3+^ and Al^3+^ (0.615 and 0.535 Å, respectively).^6^ The unit cell volumes of the investigated series change within the range from 1065.6 to 1078.9 Å^3^. Detailed unit cell parameters are presented in Table S2 and Figure S1. The comprehensive structural and vibrational studies on both EANaCr and EANaAl have been published previously.^6,7^

The phase purity of prepared materials was confirmed by XRD measurements. The collation of obtained patterns measured for the series of samples is presented in Figure S2. The increasing concentration of Cr^3+^ ions leads to a change in XRD patterns, where the most visible change takes place in a range of 20.5–21.5°. No additional diffraction lines were detected, which indicates that the Cr^3+^ ions can be substituted by the Al^3+^ ions within the full range of concentrations. The similar structural properties of the investigated materials significantly expand the field of their possible implementation.

### Raman Studies

Raman spectra of investigated compounds contain bands related to internal vibrations of HCOO^–^ and EA^+^ ions as well as lattice vibrations. The specific assignments of observed Raman bands, as well as more complex structural description for combined hybrid materials containing EA^+^ and HCOO^–^ ions of isostructural compounds, were described in the literature; thus, no particular attention will be given to this matter.^6,7^

**Figure 2:**
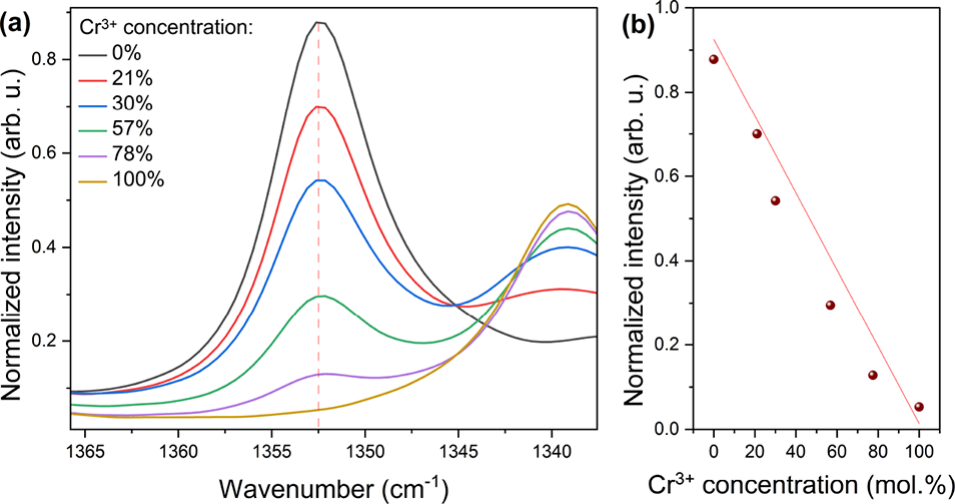
(a) Change of the normalized Raman spectra for a 1352 cm^–1^ peak corresponding to the C–H in-plane bending mode (υ_5_); (b) relation between 1352 cm^–1^ peak intensity and Cr^3+^ concentration with linear fitting.

The collation of the room-temperature Raman spectra for a series of [EA]_2_NaCr_*x*_Al_1–*x*_(HCOO)_6_ compounds is presented in Figure S3a. As one can see, the change of the composition parameter *x* affects qualitatively the spectra. The disappearance of some peaks and/or changes in intensity is accompanied by an increase in Cr^3+^ concentration. For instance, the increasing amount of Cr^3+^ causes a decrease in the intensity of bands at 227, 290, 308, 630, 936, 1352, and 1682 cm^–1^ followed by the emergence of bands at 245, 1340, 1383, and 1672 cm^–1^. Some of them strongly change the relative intensity (Figure S3b,c). These subtle differences are related to the slightly different phonon properties of the Cr^3+^/Al^3+^–O bonds and the changes in the local environments in the samples composed of the mixed CrO_6_/AlO_6_ octahedra. Additionally, a particular change is observed around 1352 cm^–1^ (Figure 2a). The variety of the peak intensities might be implemented to estimate the concentration of the Cr^3+^ ions in the sample. The exemplary intensity as a function of concentration is presented in Figure 2b. Due to the high accessibility and measurement simplicity of the Raman spectra, the calculation of the ion concentration may be a useful analytical tool for material science.^48−50^ Therefore, this topic may be an interesting subject for further investigation.

### Diffuse Reflectance Spectra

**Figure 3:**
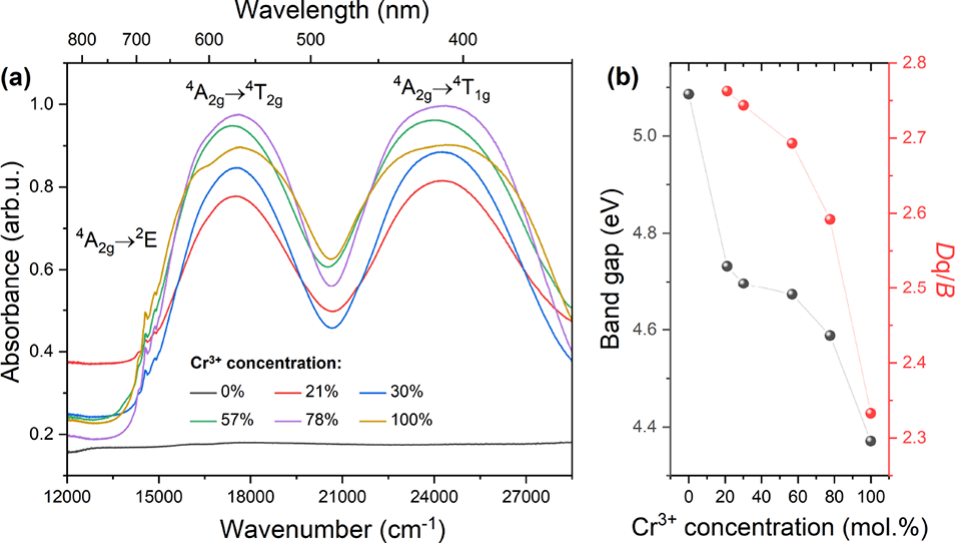
(a) Diffuse reflectance spectra of a series of [EA]_2_NaCr_*x*_Al_1–*x*_(HCOO)_6_ (*x* = 1, 0.78, 0.57, 0.30, 0.21, and 0) compounds measured at 300 K; (b) change of the energy band gap (*E*_g_) and *D*q/*B* parameters as a function of Cr^3+^ ion concentration.

The comparison of the diffuse reflectance spectra of the prepared compounds is presented in Figure 3a. A series of obtained spectra contain two main broad bands localized at about 17,452 cm^–1^ (573 nm) and 24,213 cm^–1^ (413 nm) corresponding to spin-allowed ^4^A_2g_ → ^4^T_1g_ and ^4^A_2g_ → ^4^T_2g_ transitions, respectively. Low-intensity and narrow lines at around 14,577 cm^–1^ (686 nm) are attributed to the spin-forbidden transition from the ^4^A_2g_ ground state to the ^2^E excited level. The intensity of the DRS spectrum is related to several factors, e.g., size and position of crystallites. Thus, performed measurements are used in a qualitative rather than quantitative manner. The change in the spectrum shape is caused by the decrease in spectrum components’ overlapping. In fact, each part of the spectrum assigned to ^4^A_2g_ → ^4^T_1g_ and ^4^A_2g_ → ^4^T_2g_ transitions contains two bands, in which overlapping creates the final shape of the band, which is particularly visible for a sample containing 100 mol % chromium ions.

The [EA]_2_NaAl(HCOO)_6_ sample does not exhibit absorption in the given range due to a lack of optically active chromium ions. The comprehensive description of absorption spectrum deconvolution has been described by Stręk et al.^51^

In addition, spectral changes like band broadening or maximum peak shift were observed at the absorption spectra (Figure S4).

The Kubelka–Munk function was used to calculate the energy of band gaps (*E*_g_) in the examined materials.^52^ This determination is based on the graphical examination of the following function:
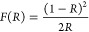
1where *R* denotes the measured diffuse reflectance. The comparison of the prepared graphical analysis is presented in Figure S5a–f. The estimated values of band gaps are presented in Figure 3b. The nonlinear decrease in *E*_g_ value is observed across the entire range of Cr^3+^ concentrations due to the substitution of the smaller Al^3+^ ion (0.535) by the larger Cr^3+^ (0.615) one. The sample containing only Al^3+^ ions exhibits the maximum value of the band gap energy (5.09 eV), whereas the compound based only on Cr^3+^ ions shows a minimum value of 4.38 eV. The reduction of the *E*_g_ value due to an increase in Cr^3+^ ion concentration has been reported for similar compounds containing a methylammonium cation.^14^

**Figure 4:**
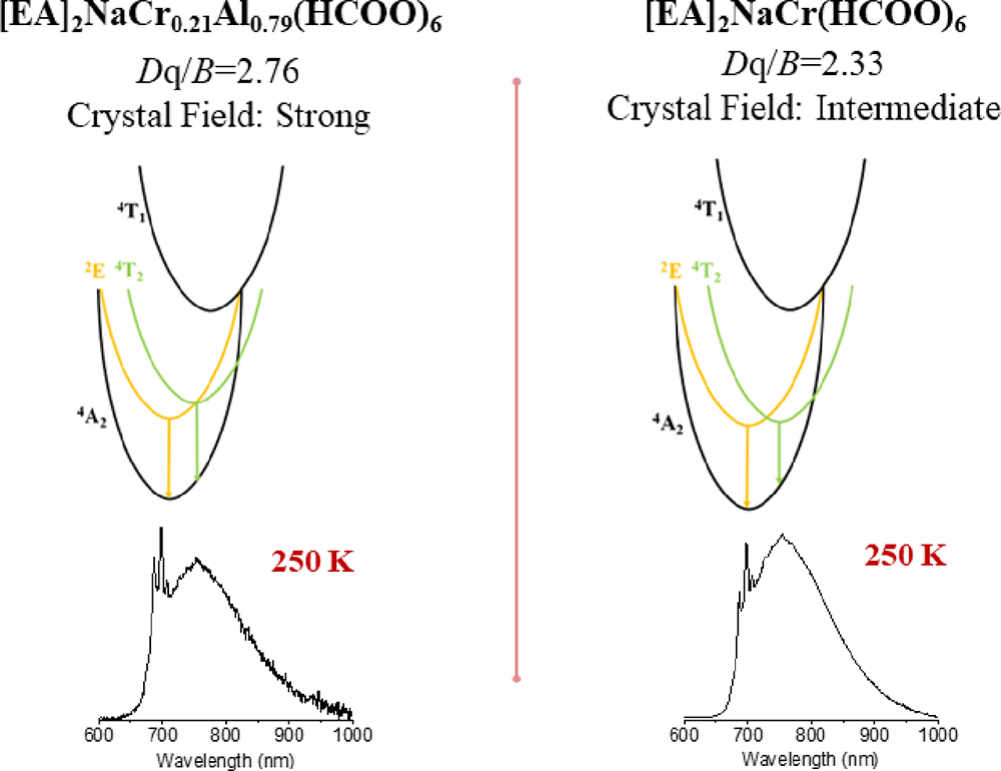
Comparison of the *D*q/*B* values of two samples containing 21 and 100 mol % Cr^3+^ ions as well as energy diagrams of Cr^3+^ ions with the obtained emission spectra (normalized).

The obtained diffuse reflectance spectra were used to determine the crystal field (*D*q), as well as the Racah parameters (*B* and *C*). The calculations were conducted following the previously reported methodology.^16,22^ The detailed procedure of crystal field parameter determination is presented in Instruction 1 (Supporting Information). The summary of the calculation values is presented in the Supporting Information (Table S3). The analysis of the *D*q/*B* ratio allows the determination of the crystal field strength. According to the Tanabe–Sugano diagram, the ^2^E_g_ and ^4^T_2g_ levels are overlapped for the *D*q*/B* ratio equal to 2.3, which separates strong (*D*q*/B* > 2.3) and weak (*D*q*/B* < 2.3) crystal fields. Additionally, samples exhibiting a *D*q/*B* value close to 2.3 can be assigned to the so-called intermediate crystal field. The sample containing the lowest concentration of Cr^3+^ ions (21 mol %) exhibits the strongest crystal field (*D*q*/B* = 2.76). The increasing concentration of Cr^3+^ ions leads to a reduction of the *D*q*/B* value (Figure 3b). The lowest value of the *D*q*/B* parameter (2.33) was calculated for the sample containing 100 mol % chromium ions. Thus, the series of investigated compounds can be mainly described as exhibiting strong crystal fields. However, the sample containing 100% Cr^3+^ ions exhibits an intermediate crystal field. The comparison of two samples containing 21 and 100 mol % Cr^3+^ with energy diagrams and representative emission spectra is presented in Figure 4. The obtained results confirm the crystal field strength reduction by increasing the chromium concentration, which was reported previously.^14^

### Luminescence and Temperature Dependency

The emission spectra of the obtained compounds were measured within the range of 80–400 K with 10 K steps. The used excitation wavelength was 405 nm since this energy corresponds well with the ^4^A_2g_ → ^4^T_1g_ transition. The collation of the excitation and emission spectra of [EA]_2_NaCr_0.78_Al_0.22_(HCOO)_6_ is presented in Figure S6. Due to fast nonradiative relaxation, the energy transfer from ^4^T_1g_ to ^4^T_2g_ and ^2^E_g_ levels occurs.^6^ The emission spectra of investigated compounds are strongly sensitive to the environmental temperature. At low temperatures, several narrow bands are present, where the strongest one is located at 686.4 nm (named the R_1_ line). Moreover, the band at 684.2 nm (named R_2_) can be observed. There are also additional Stokes bands localized at 696.8, 706.5, 727.2, and 752.3 nm. At higher temperatures, the intensities of the R_1_ and R_2_ bands significantly decrease. The intensities of bands at 727.2 and 752.3 nm also simultaneously decrease, although this process is not as progressive as a reduction of more intensive bands.

As the temperature increases, the emission spectra expand and create a wide band with a maximum at 752.3 nm. A progressive increase in temperature leads to a reduction of the emission intensity. The maximum intensities of this band occur at 210, 190, 185, and 150 K for samples containing 100, 78, 57, and 21 mol % chromium ions, respectively.

This emission is assigned to the spin-allowed ^4^T_2g_ → ^4^A_2g_ transitions. The formation of this broad band depends on the concentration of the Cr^3+^ ions. Therefore, the sample of [EA]_2_NaCr_0.21_Al_0.79_(HCOO)_6_ does not exhibit such a property and, on the other hand, the sample of [EA]_2_NaCr(HCOO)_6_ shows the strongest emission from the ^4^T_2g_ level.

The luminescence properties of transition metal ions, such as Cr^3+^, originate from d–d electronic transitions, which are not shielded by the outer orbitals, unlike the 4f orbitals in trivalent lanthanide ions.^53^ Consequently, the spectroscopic characteristics of Cr^3+^ ions are strongly affected by the crystal field strength of the matrix material; thus, the emission type, range, and thermal quenching rate can be tuned by changing the matrix type.^53−56^ The sensitivity of spectroscopic properties to the change of crystal field parameters plays a particular role in research on TM-based luminescence thermometers.^13,14,57^ The irradiation with 405 nm wavelength excites a higher ^4^T_1g_ level. The nonradiative energy transfer causes the increase in the population of the lowest vibrational level of ^2^E_g_. At low temperatures, the ^2^E_g_ → ^4^A_2g_ transition is dominant. An increase in temperature leads to a thermal population of the ^4^T_2g_ level, which causes the occurrence of the broad spin-allowed ^4^T_2g_ → ^4^A_2g_ transition. The emission of the Cr^3+^ ions may be influenced by the local ion’s symmetry changes, which causes a distortion of the excited state parabola.^58^ Due to the occurrence of the crossing point between ground and excitation state parabolas, the increasing temperature may cause the depopulation of the excitation states via nonradiative transitions.

The same behavior was previously reported in methylammonium-based materials exhibiting different crystal field properties, particularly the *D*q/*B* parameter.^14^ The overall emission of prepared compounds quenches rapidly due to an increase in temperature to around 300 K. The concentration of Cr^3+^ ions affects the maximal temperature when any emission is detectable, which is 270 and 330 K for samples containing 21 and 100 mol % Cr^3+^ ions, respectively.

It is worth noting that the sample of [EA]_2_NaCr(HCOO)_6_ exhibits low intensity and a very broad band within a range of 770 to 870 nm at a low temperature (80–130 K). This behavior was not detected in samples containing a lower concentration of Cr^3+^ ions. Such an occurrence of the additional band may indicate surface defects that can be related to a high concentration of chromium ions. A similar additional emission peak was reported for hybrid perovskites, e.g., CH_3_NH_3_PbCl_3_ or MHyPbCl_3_, and it has been assigned to the recombination of photoexcited carriers in defects.^59,60^

**Figure 5:**
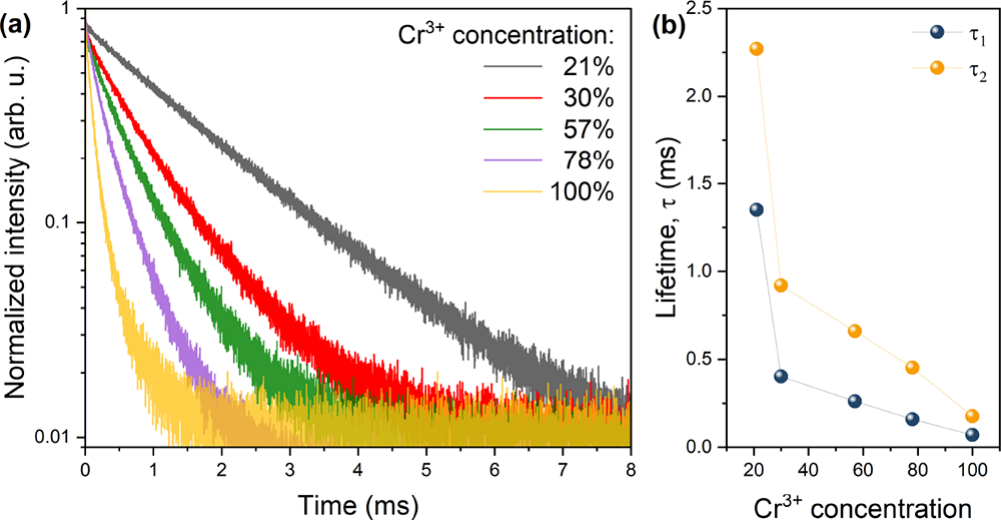
(a) Decay profiles of [EA]_2_NaCr_*x*_Al_1–*x*_(HCOO)_6_ (*x* = 1, 0.78, 0.57, 0.30, and 0.21) measured at 77 K; (b) change of time parameters as a function of Cr^3+^ concentration.

Additionally, the decay profiles for samples containing 21–100 mol % Cr^3+^ were measured (Figure 5a). To calculate the time components of the decay (τ_1_ and τ_2_ parameters), double exponential fitting was performed with the following equation:
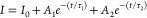
2where *I*_0_ is the initial luminescence intensity, *A*_1_ and *A*_2_ are the pre-exponential coefficients, τ_1_ and τ_2_ are first and second time components, respectively, and *t* is the time.

The influence of the chromium ions on the decay curve shape is observed. The highest values of τ_1_ and τ_2_ were calculated for the [EA]_2_NaCr_0.21_Al_0.79_(HCOO)_6_ sample and were equal to 1.35 and 2.26 ms, respectively. The increase in chromium ion concentration leads to the nonlinear reduction of the τ_1_ and τ_2_ parameters (Figure 5b). The most significant decrease occurs between 21 and 30 mol % Cr^3+^ ion concentrations, and then the change of the time parameters is closer to linear. The lowest values of τ_1_ (0.069 ms) and τ_2_ (0.176 ms) were calculated for the [EA]_2_NaCr(HCOO)_6_ sample. The values of the time parameters for all of the measured samples are listed in Table S4.

### Luminescence Thermometry

The observed changes in the intensity and character of the emission in the aftermath of temperature change make the investigated metal–organic frameworks interesting materials for luminescence thermometry. It was recently suggested that hybrid materials with a perovskite-type architecture containing Cr^3+^ ions exhibit a sufficient optical response and physicochemical properties to be implemented into noncontact temperature sensing solutions.^13,45,46,57,61^

**Figure 6:**
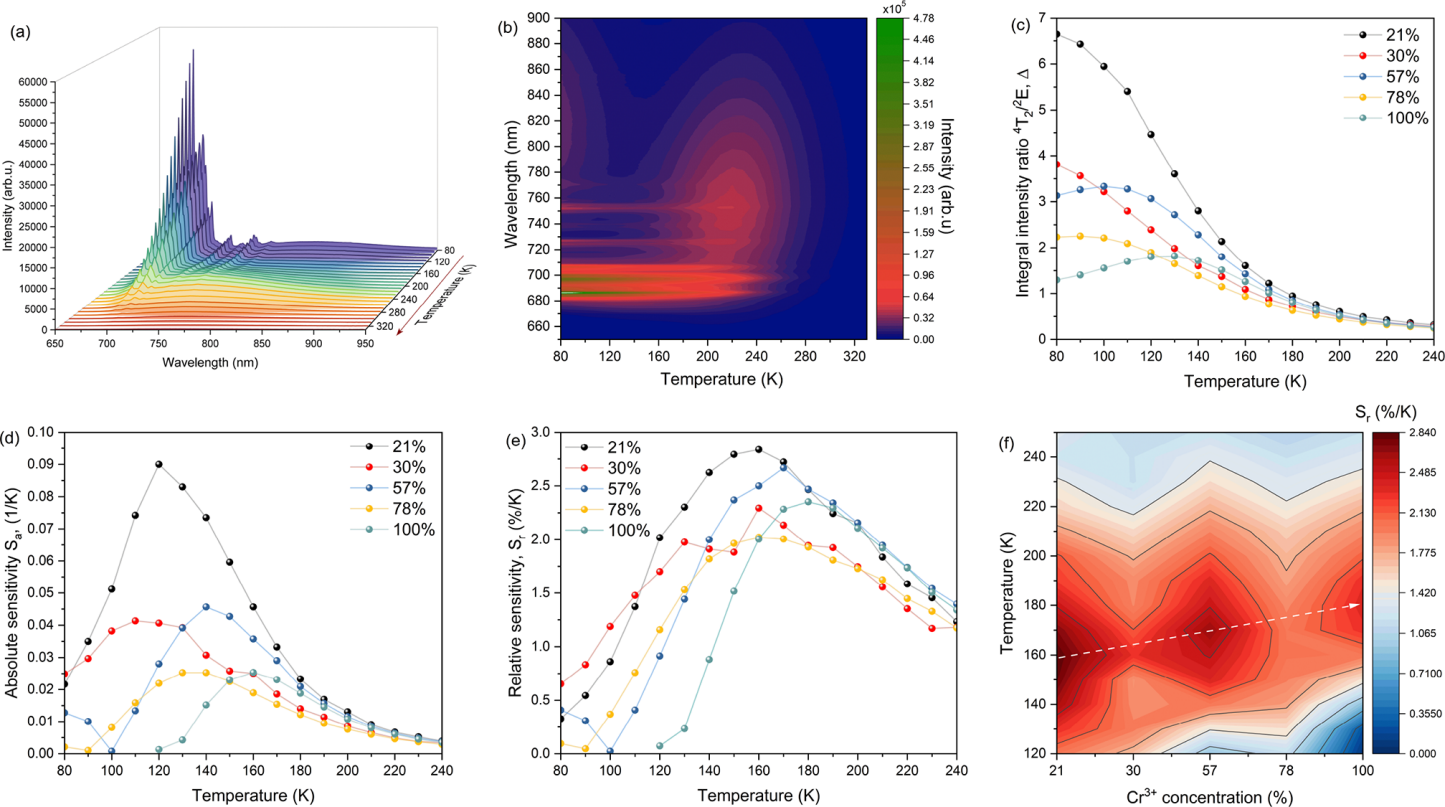
(a) Temperature-dependent emission spectra and (b) thermal evolution of the intensity measured for the [EA]_2_NaCr(HCOO)_6_ sample; (c) thermometric parameter (Δ); (d) absolute sensitivity (*S*_a_); (e) relative sensitivity (*S*_r_) and (f) influence of the Cr^3+^ ion concentration and temperature on the relative sensitivity.

Temperature sensing originates from the coexistence of at least two temperature-dependent transitions. In the case of [EA]_2_NaCr_*x*_Al_1–*x*_(HCOO)_6_ compounds, the change in intensities of two bands, assigned to ^4^T_2g_ → ^4^A_2g_ and ^2^E_g_ → ^4^A_2g_, serves as the basis for temperature estimation. The thermal evolution of the emission spectra for the sample containing 100 mol % Cr^3+^ is presented in Figure 6a,b. The results for the remaining samples are presented in Figure S7. To demonstrate the temperature sensing performance, the thermometric parameter Δ (FIR) was calculated. It is described as the ratio of the integrated intensities of the considered bands. For the calculations, two regions were chosen, namely, 660–718 nm for ^2^E_g_ → ^4^A_2g_ (denoted as *I*_1_) and 718–970 nm for ^4^T_2g_ → ^4^A_2g_ (denoted as *I*_2_). The comparison of the Δ value as a function of the temperature for a series of investigated materials is presented in Figure 6c.

The increase in temperature causes the general reduction of Δ; however, for samples containing 100 and 57 mol % Cr^3+^ ions, the increase in the calculated value is observed within ranges of 80–130 and 80–100 K, respectively. The highest initial value of Δ was calculated for a sample containing 21 mol % Cr^3+^. Within a range of 200–300 K, the calculated values of the thermometric parameters for all samples are comparable. The slightly different shape of the initial part of the plot for the material containing 100% Cr^3+^ ions may be related to the additional, low-intensity broad band associated with a possible self-trapped exciton (STE), whose existence was reported for a wide variety of perovskite materials.^62−64^ The observed emission decreases rapidly due to the increase in temperature.

To further demonstrate the luminescence thermometry performance, the absolute (*S*_a_) and relative (*S*_r_) sensitivities were calculated. The parameters are described by the following equations:^13^
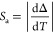
3and
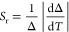
4where dΔ represents the change of thermometer parameter Δ (Figure 6c) at temperature change d*T*. The collations of absolute sensitivities for a series of investigated materials are presented in Figure 6d. The absolute sensitivities initially increase with the growth of temperature. The maximum value of *S*_a_ is 0.09 K^–1^ at 120 K for a sample containing 21 mol % Cr^3+^ ions. A progressive increase in temperature causes a significant decrease in *S*_a_ value. Similarly, the relative sensitivity values increase with temperature (Figure 6e). The maximum value of *S*_r_ is 2.84% K^–1^ and is reached again for a sample containing 21% chromium ions at a temperature of 160 K. The calculated temperature measurement uncertainty (δ*T*) for the [EA]_2_NaCr_0.21_Al_0.79_(HCOO)_6_ sample at 160 K was 0.40 K. An increasing temperature leads to a simultaneous decrease in *S*_r_ parameter. The final value of relative sensitivity for most of the samples is 1.04–1.33% K^–1^. In addition, the dependency of chromium ion concentration on the useful temperature sensing range and relative sensitivity can be observed. The increasing concentration of Cr^3+^ ions causes the change of the maximal relative sensitivity toward a higher temperature (Figure 6f). The possible modulation of the optimal sensing range by tuning the Cr^3+^ concentration significantly expands the possibility of system optimization.

**Figure 7:**
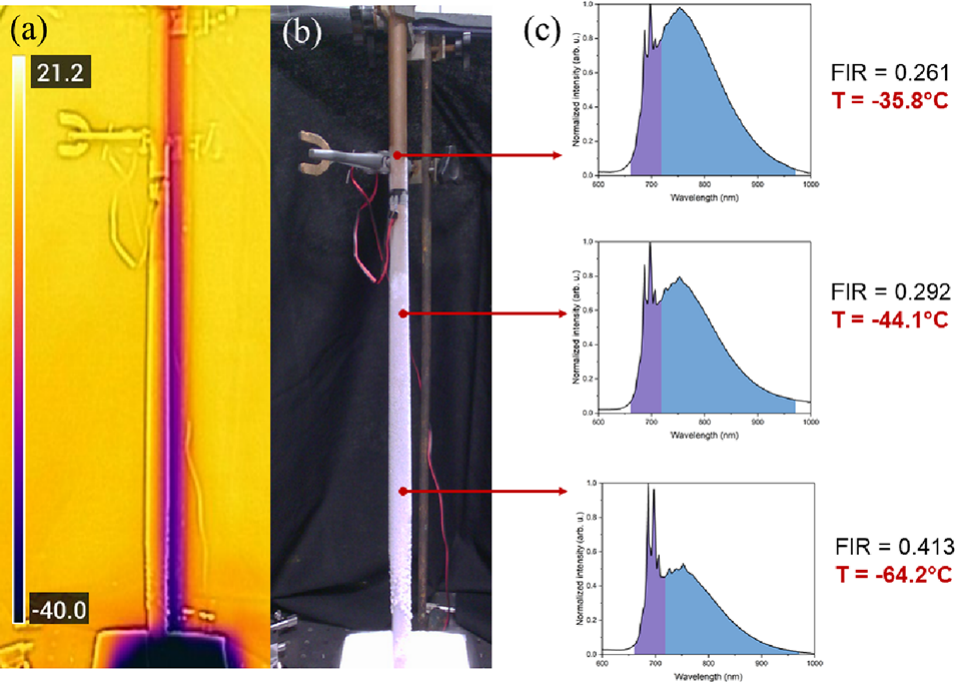
Exemplary thermometric system: (a) thermal image and (b) picture of the experimental setup; (c) recorded emission spectra for individual points with calculated FIR.

To initially demonstrate a practical potential of luminescence thermometry, the exemplary thermometric solution based on one of presented compounds has been prepared (Figure 7). The obtained materials were investigated as luminescence thermometers for low-temperature (*T* < −30 °C) systems. Luminescence thermometers have to be cooled by contact with an object, whose temperature is to be measured. The very first tests were performed with the system based on the temperature gradient present in a copper pipe, one of which end is submerged in liquid nitrogen (Figure 7a,b). Crystals of [EA]_2_NaCr(HCOO)_6_ were attached to the surface of the pipe with a thermal paste.

Thermometric calculations were performed with the model determined with presented temperature-dependent luminescence properties. The obtained photoluminescence spectra (λ_exc._ = 405 nm) were used to calculate the integrated intensity ratio (FIR parameter) and then compared to the model temperature relation (Figure 7c).

The presented result shows the undeniable potential of temperature-sensitive luminescent materials. Among various advantages, one of the most significant is the possibility to record the spectrum even in a presence of hoar frost. In addition, even expensive commercially available thermal imaging cameras are able to operate mainly in a temperature regime above −40 °C. The implementation of functional and durable luminescence thermometers is a significant matter for more developed investigation. Investigated organic–inorganic phosphors exhibit sufficient stability during heating–cooling intervals (Figure S8). Performed measurements consist of several consecutive heating and cooling processes. A significant change in the FIR value has not been observed, so the materials can be reused in thermometric systems. Measured materials do not exhibit phase transitions within the thermometric range, which additionally improves the stability in nonconstant temperature conditions. It is worth noting that possible practical usefulness may be significantly improved by protecting solutions, such as resin impregnation.

The obtained results, especially considering the luminescence thermometry performance, are remarkable and provide valuable information about the relationship between the composition, crystal field strength, and optical properties. Achieved values of relative sensitivity, up to 2.84% K^–1^, are comparable to conventional inorganic lanthanide-doped materials.^13,65−67^ The comparison of the *S*_r_ parameters of some reported luminescence thermometers is shown in Table 1.

**Table 1:** Collation of Exemplary Luminescence Thermometers with Relative Sensitivity (*S*_r_) at a Given Temperature (*T*)^a^

compound	*S*_r_(% K^–1^)	*T* (K)	ref.
Sr_2_MgAl_22_O_36_:Cr^3+^	1.7	310	(46)
La_2_MgTiO_6_:Cr^3+^, V^4+^	1.96	165	(13)
La_2_MgTiO_6_:Eu^3+^	3.0	77	(65)
SrGdLiTeO_6_:Mn^4+^, Eu^3+^	4.9	550	(68)
Li_5_Zn_8_Al_5_Ge_9_O_36_:Mn^2+^	8.489	323	(69)
Sr_2_GeO_4_:Pr^3+^	9.0	22	(70)
ZnGa_2_O_4_:Cr^3+^	2.8	310	(45)
[EA]_2_NaCr_0.21_Al_0.79_(HCOO)_6_	2.84	160	this work
Tb0.9Eu0.1(pia)	3.27	300	(71)
Tb_0.95_Eu_0.05_(btb)	2.85	14	(72)
Tb_0.957_Eu_0.043_cpda	16.0	300	(73)
FIR-8⊂DMASM	2.98	341	(74)

a H2pia, 5-(pyridin-4-yl)isophthalic acid; H3btb, 3,5-tris(4-carboxyphenyl)benzene; H3cpda, 5-(4-carboxyphenyl)-2,6-pyridinedicarboxylic acid; DMASM, 4-[*p*-(dimethylamino)styryl]-1-methylpyridinium.

The results show that formate-based hybrid compounds with the perovskite-like architecture [EA]_2_NaCr_*x*_Al_1–*x*_(HCOO)_6_ are promising materials for luminescence thermometry. The performed analysis shows that low-concentration chromium-doped materials may be promising compounds for highly sensitive and lanthanide-free temperature sensors.

## Conclusions

The microwave-assisted solvothermal method was used to successfully synthesize a series of MOFs with the general formula [EA]_2_NaCr_*x*_Al_1–*x*_(HCOO)_6_, where *x* = 1, 0.78, 0.57, 0.30, 0.21, and 0. XRD measurements confirmed the phase purity and the ability to obtain mixed structures across the whole concentration range. The Raman spectra confirmed the expected composition of the prepared materials and provided preliminary information on how the phonon properties change as the Cr^3+^ concentration increases. The emission properties of synthesized compounds were found to be strongly temperature-dependent below 250 K. This feature and the coexistence of temperature-sensitive bands, spin-forbidden ^2^E_g_ → ^4^A_2g_ and spin-allowed ^4^T_2g_ → ^4^A_2g_, let one to perform calculations in order to evaluate the possible implementation of investigated materials as luminescence thermometers. The relative and absolute sensitivities of the studied compounds are satisfactory and are comparable to those of known inorganic materials. The highest relative sensitivity (2.84% K^–1^, δ*T* = 0.40 K) was achieved for a sample of [EA]_2_NaCr_0.21_Al_0.79_(HCOO)_6_ at 160 K. The results also showed that the concentration of Cr^3+^ ions has a big impact on luminescence outputs. The temperature sensing range and temperature of the maximal relative sensitivity can be precisely tuned by modifying the sample composition, substantially increasing the utility of the developed luminescence thermometer. According to the results of the investigation, chromium-based organic–inorganic perovskites could be promising materials for noncontact temperature sensing. The presented practical implementation of the investigated compounds shows the potential of this particular type of sensor. The development of thermosensitive materials containing transition metal ions could pave the way for lanthanide-free, low-cost, and efficient contactless luminescence thermometry solutions.

## Supporting Information Available

Quantities of precursors; unit cell parameters; crystal field parameters and electron transition energies; time parameters of decays; change of unit cell parameters; XRD; Raman spectra; normalized DRS spectra; band gap determined with the Kubelka–Munk function; excitation–emission spectra; temperature dependence on the luminescence spectra; thermometric stability in 100–160 K intervals for the representative sample; thermal stability and crystal field parameter calculation (PDF)

## Notes

Experimental data: The Raman and diffuse reflectance spectra, band gap and crystal field strength, decay profiles and lifetimes, temperature-dependent luminescence and emission maps, thermometric parameters, exemplary system’s luminescence characteristics, unit cell characteristics, powder XRD data, Kubelka–Munk function with band gap estimation, excitation and emission spectra, and stability of PL are available at 10.5281/zenodo.7505524.

## References

[ref1] Ptak M.; Sieradzki A.; Šimėnas M.; Mączka M. Molecular Spectroscopy of Hybrid Organic–Inorganic Perovskites and Related Compounds. Coord. Chem. Rev. 2021, 448, 213180 10.1016/j.ccr.2021.214180.

[ref2] Fan Z.; Sun K.; Wang J. Perovskites for Photovoltaics: A Combined Review of Organic-Inorganic Halide Perovskites and Ferroelectric Oxide Perovskites. J. Mater. Chem. A 2015, 3, 18809–18828. 10.1039/c5ta04235f.

[ref3] Prochowicz D.; Franckevičius M.; Cieslak A. M.; Zakeeruddin S. M.; Gratzel M.; Lewinski J. Mechanosynthesis of the Hybrid Perovskite CH_3_NH_3_PbI_3_: Characterization and the Corresponding Solar Cell Efficiency. J. Mater. Chem. A 2015, 3, 20772–20777. 10.1039/c5ta04904k.

[ref4] Kim J. Y.; Lee J. W.; Jung H. S.; Shin H.; Park N. G. High-Efficiency Perovskite Solar Cells. Chem. Rev. 2020, 120, 7867–7918. 10.1021/acs.chemrev.0c00107. 32786671

[ref5] Huang C. R.; Luo X.; Chen X. G.; Song X. J.; Zhang Z. X.; Xiong R. G. A Multiaxial Lead-Free Two-Dimensional Organic-Inorganic Perovskite Ferroelectric. Natl. Sci. Rev. 2021, 8, 1–7. 10.1093/nsr/nwaa232. PMC8288432 34691638

[ref6] Ptak M.; Mączka M.; Gągor A.; Sieradzki A.; Bondzior B.; Dereń P.; Pawlus S. Phase Transitions and Chromium(III) Luminescence in Perovskite-Type [C_2_H_5_NH_3_][Na_0. 5_Cr_x_Al_0.5- x_(HCOO)_3_] (x = 0, 0.025, 0.5), Correlated with Structural, Dielectric and Phonon Properties. Phys. Chem. Chem. Phys. 2016, 18, 29629–29640. 10.1039/c6cp05151k. 27752659

[ref7] Ptak M.; Mączka M.; Gągor A.; Sieradzki A.; Stroppa A.; di Sante D.; Perez-Mato J. M.; MacAlik L. Experimental and Theoretical Studies of Structural Phase Transition in a Novel Polar Perovskite-like [C_2_H_5_NH_3_][Na_0. 5_Fe_0.5_(HCOO)_3_] Formate. Dalton Trans. 2016, 45, 2574–2583. 10.1039/c5dt04536c. 26725595

[ref8] Wang Z. C.; Rogers J. D.; Yao X.; Nichols R.; Atay K.; Xu B.; Franklin J.; Sochnikov I.; Ryan P. J.; Haskel D.; Tafti F. Colossal Magnetoresistance without Mixed Valence in a Layered Phosphide Crystal. Adv. Mater. 2021, 33, 2005755–2005762. 10.1002/adma.202005755. 33511677

[ref9] Zhang J.; Ji W.-J.; Xu J.; Geng X.-Y.; Zhou J.; Gu Z.-B.; Yao S.-H.; Zhang S.-T. Giant Positive Magnetoresistance in Half-Metallic Double-Perovskite Sr_2_CrWO_6_ Thin Films. Sci. Adv. 2017, 3, 1–7. 10.1126/sciadv.1701473. PMC5669608 29119138

[ref10] Dhahri A.; Dhahri E.; Hlil E. K. Large Magnetocaloric Effect in Manganese Perovskite La_0. 67–x_Bi_x_Ba_0.33_MnO_3_ near Room Temperature. RSC Adv. 2019, 9, 5530–5539. 10.1039/c8ra09802f. 35515922 PMC9060775

[ref11] Kadim G.; Masrour R.; Jabar A.; Hlil E. K. Room-Temperature Large Magnetocaloric, Electronic and Magnetic Properties in La_0.75_Sr_0.25_MnO_3_ Manganite: Ab Initio Calculations and Monte Carlo Simulations. Phys. A 2021, 573, 125936 10.1016/j.physa.2021.125936.

[ref12] Chen C. W.; Hsiao S. Y.; Chen C. Y.; Kang H. W.; Huang Z. Y.; Lin H. W. Optical Properties of Organometal Halide Perovskite Thin Films and General Device Structure Design Rules for Perovskite Single and Tandem Solar Cells. J. Mater. Chem. A 2015, 3, 9152–9159. 10.1039/c4ta05237d.

[ref13] Stefańska D.; Bondzior B.; Vu T. H. Q.; Grodzicki M.; Dereń P. J. Temperature Sensitivity Modulation through Changing the Vanadium Concentration in a La_2_MgTiO_6_:V^5+^,Cr^3+^ Double Perovskite Optical Thermometer. Dalton Trans. 2021, 50, 9851–9857. 10.1039/d1dt00911g. 34195737

[ref14] Ptak M.; Dziuk B.; Stefańska D.; Hermanowicz K. The Structural, Phonon and Optical Properties of [CH_3_NH_3_]M_0. 5_Cr: XAl_0.5- x_(HCOO)_3_ (M = Na, K; X = 0, 0.025, 0.5) Metal-Organic Framework Perovskites for Luminescence Thermometry. Phys. Chem. Chem. Phys. 2019, 21, 7965–7972. 10.1039/c9cp01043b. 30924483

[ref15] Zhu H.; Fu Y.; Meng F.; Wu X.; Gong Z.; Ding Q.; Gustafsson M. V.; Trinh M. T.; Jin S.; Zhu X. Y. Lead Halide Perovskite Nanowire Lasers with Low Lasing Thresholds and High Quality Factors. Nat. Mater. 2015, 14, 636–642. 10.1038/nmat4271. 25849532

[ref16] Ptak M.; Stefańska D.; Gągor A.; Svane K. L.; Walsh A.; Paraguassu W. Heterometallic Perovskite-Type Metal–Organic Framework with an Ammonium Cation: Structure, Phonons, and Optical Response of [NH_4_]Na_0.5_CrxAl_0.5_-x(HCOO)_3_ (X= 0, 0.025 and 0.5). Phys. Chem. Chem. Phys. 2018, 20, 22284–22295. 10.5281/zenodo.1289169. 30123897

[ref17] Zhang Y.; Liao W. Q.; Fu D. W.; Ye H. Y.; Liu C. M.; Chen Z. N.; Xiong R. G. The First Organic-Inorganic Hybrid Luminescent Multiferroic: (Pyrrolidinium)MnBr_3_. Adv. Mater. 2015, 27, 3942–3946. 10.1002/adma.201501026. 26011784

[ref18] Shang R.; Xu G. C.; Wang Z. M.; Gao S. Phase Transitions, Prominent Dielectric Anomalies, and Negative Thermal Expansion in Three High Thermally Stable Ammonium Magnesium-Formate Frameworks. Chem. – Eur. J. 2014, 20, 1146–1158. 10.1002/chem.201303425. 24375515

[ref19] Mączka M.; Ptak M.; Pawlus S.; Paraguassu W.; Sieradzki A.; Balciunas S.; Simenas M.; Banys J. Temperature- and Pressure-Dependent Studies of Niccolite-Type Formate Frameworks of [NH_3_(CH_2_)_4_NH_3_][M_2_(HCOO)_6_] (M = Zn, Co, Fe). Phys. Chem. Chem. Phys. 2016, 18, 27613–27622. 10.1039/c6cp05834e. 27711614

[ref20] Xu X. Y.; Yan B. An Efficient and Sensitive Fluorescent PH Sensor Based on Amino Functional Metal-Organic Frameworks in Aqueous Environment. Dalton Trans. 2016, 45, 7078–7084. 10.1039/c6dt00361c. 27002862

[ref21] Cui Y.; Zhu F.; Chen B.; Qian G. Metal-Organic Frameworks for Luminescence Thermometry. Chem. Commun. 2015, 51, 7420–7431. 10.1039/c5cc00718f. 25715078

[ref22] Mączka M.; Bondzior B.; Dereń P.; Sieradzki A.; Trzmiel J.; Pietraszko A.; Hanuza J. Synthesis and Characterization of [(CH_3_)_2_NH_2_][Na_0. 5_Cr_0.5_(HCOO)_3_]: A Rare Example of Luminescent Metal-Organic Frameworks Based on Cr(III) Ions. Dalton Trans. 2015, 44, 6871–6879. 10.1039/c5dt00060b. 25773714

[ref23] Ptak M.; Zarychta B.; Stefańska D.; Ciupa A.; Paraguassu W. Novel Bimetallic MOF Phosphors with an Imidazolium Cation: Structure, Phonons, High- Pressure Phase Transitions and Optical Response. Dalton Trans. 2019, 48, 242–252. 10.1039/C8DT04246B. 30516206

[ref24] Charroux B.; Daian F.; Royet J. Drosophila Aversive Behavior toward Erwinia carotovora carotovora Is Mediated by Bitter Neurons and Leukokinin. iScience 2020, 23, 101152 10.1016/j.isci.2020.101152. 32450516 PMC7251953

[ref25] Dereń P. J.; Malinowski M.; Strȩk W. Site Selection Spectroscopy of Cr^3+^ in MgA1204 Green Spinel. J. Lumin. 1996, 68, 91–103. 10.1016/0022-2313(96)00020-8.

[ref26] Deren P. J.; Watras A.; Gagor A.; Pazik R. Weak Crystal Field in Yttrium Gallium Garnet (YGG) Submicrocrystals Doped with Cr 3+. Cryst. Growth Des. 2012, 12, 4752–4757. 10.1021/cg300435t.

[ref27] Wang Q.; Liao M.; Lin Q.; Xiong M.; Mu Z.; Wu F. A Review on Fluorescence Intensity Ratio Thermometer Based on Rare-Earth and Transition Metal Ions Doped Inorganic Luminescent Materials. J. Alloys Compd. 2021, 850, 156744 10.1016/j.jallcom.2020.156744.

[ref28] Yuan J.; Zhang Y.; Xu J.; Tian T.; Luo K.; Huang L. Novel Cr^3+^-Doped Double-Perovskite Ca_2_MNbO_6_ (M = Ga, Al) Phosphor: Synthesis, Crystal Structure Photoluminescence and Thermoluminescence Properties. J. Alloys Compd. 2020, 815, 152656 10.1016/j.jallcom.2019.152656.

[ref29] Lin H.; Bai G.; Yu T.; Tsang M. K.; Zhang Q.; Hao J. Site Occupancy and Near-Infrared Luminescence in Ca_3_Ga_2_Ge_3_O_12_: Cr^3+^ Persistent Phosphor. Adv. Opt. Mater. 2017, 5, 1700227 10.1002/adom.201700227.

[ref30] Marciniak L.; Bednarkiewicz A. Nanocrystalline NIR-to-NIR Luminescent Thermometer Based on Cr^3+^, Yb^3+^ Emission. Sens. Actuators, B 2017, 243, 388–393. 10.1016/j.snb.2016.12.006.

[ref31] Maturi F. E.; Brites C. D. S.; Ximendes E. C.; Mills C.; Olsen B.; Jaque D.; Ribeiro S. J. L.; Carlos L. D. Going Above and Beyond: A Tenfold Gain in the Performance of Luminescence Thermometers Joining Multiparametric Sensing and Multiple Regression. Laser Photonic Rev. 2021, 15, 2100301 10.1002/lpor.202100301.

[ref32] Yin H. Q.; Yin X. B. Metal-Organic Frameworks with Multiple Luminescence Emissions: Designs and Applications. Acc. Chem. Res. 2020, 53, 485–495. 10.1021/acs.accounts.9b00575. 31999097

[ref33] Wu S.; Min H.; Shi W.; Cheng P. Multicenter Metal–Organic Framework-Based Ratiometric Fluorescent Sensors. Adv. Mater. 2020, e1805871 10.1002/adma.201805871. 30790371

[ref34] N’Dala-Louika I.; Ananias D.; Latouche C.; Dessapt R.; Carlos L. D.; Serier-Brault H. Ratiometric Mixed Eu-Tb Metal-Organic Framework as a New Cryogenic Luminescent Thermometer. J. Mater. Chem. C 2017, 5, 10933–10937. 10.1039/c7tc03223d.

[ref35] del Rosal B.; Ximendes E.; Rocha U.; Jaque D. In Vivo Luminescence Nanothermometry: From Materials to Applications. Adv. Opt. Mater. 2017, 5, 1600508 10.1002/adom.201600508.

[ref36] Dramićanin M. D. Trends in Luminescence Thermometry. J. Appl. Phys. 2020, 128, 040902 10.1063/5.0014825.

[ref37] Brites C. D. S.; Millán A.; Carlos L. D. Lanthanides in Luminescent Thermometry. In Handbook on the Physics and Chemistry of Rare Earths; Elsevier B.V., 2016; Vol. 49, pp. 339–427. 10.1016/bs.hpcre.2016.03.005.

[ref38] Rocha J.; Brites C. D.; Carlos L. D. Lanthanide Organic Framework Luminescent Thermometers. Chem. – Eur. J. 2016, 22, 14782–14795. 10.1002/chem.201600860. 27482637

[ref39] Kolesnikov I. E.; Afanaseva E. V.; Kurochkin M. A.; Kolesnikov E. Y.; Lähderanta E. Mixed-Valent MgAl_2_O_4_:Eu^2+^/Eu^3+^ Phosphor for Ratiometric Optical Thermometry. Phys. B 2022, 624, 413456 10.1016/j.physb.2021.413456.

[ref40] Trojan-Piegza J.; Brites C. D. S.; Ramalho J. F. C. B.; Wang Z.; Zhou G.; Wang S.; Carlos L. D.; Zych E. La_0. 4_Gd_1.6_Zr_2_O_7_:0.1%Pr Transparent Sintered Ceramic - a Wide-Range Luminescence Thermometer. J. Mater. Chem. C 2020, 8, 7005–7011. 10.1039/d0tc00861c.

[ref41] Gavrilović T. V.; Jovanović D. J.; Lojpur V.; Dramićanin M. D. Multifunctional Eu^3+^- and Er^3+^/Yb^3+^-Doped GdVO_4_ Nanoparticles Synthesized by Reverse Micelle Method. Sci. Rep. 2014, 4, 4209 10.1038/srep04209. 24572638 PMC3936229

[ref42] Mykhaylyk V.; Kraus H.; Zhydachevskyy Y.; Tsiumra V.; Luchechko A.; Wagner A.; Suchocki A. Multimodal Non-Contact Luminescence Thermometry with Cr-Doped Oxides. Sensors 2020, 20, 1–22. 10.3390/s20185259. PMC7570664 32942602

[ref43] Glais E.; Pellerin M.; Castaing V.; Alloyeau D.; Touati N.; Viana B.; Chanéac C. Luminescence Properties of ZnGa_2_O_4_ :Cr^3+^, Bi^3+^ Nanophosphors for Thermometry Applications. RSC Adv. 2018, 8, 41767–41774. 10.1039/c8ra08182d. 35558763 PMC9091948

[ref44] Elzbieciak K.; Marciniak L. The Impact of Cr^3+^ Doping on Temperature Sensitivity Modulation in Cr^3+^ Doped and Cr^3+^, Nd^3+^ Co-Doped Y_3_Al_5_O_12_, Y_3_Al_2_Ga_3_O_12_, and Y_3_Ga_5_O_12_ Nanothermometers. Front. Chem. 2018, 6, 1–8. 10.3389/fchem.2018.00424. 30283774 PMC6157326

[ref45] Ueda J.; Back M.; Brik M. G.; Zhuang Y.; Grinberg M.; Tanabe S. Ratiometric Optical Thermometry Using Deep Red Luminescence from ^4^T_2_ and ^2^E States of Cr^3+^ in ZnGa_2_O_4_ Host. Opt. Mater. 2018, 85, 510–516. 10.1016/j.optmat.2018.09.013.

[ref46] Wang Q.; Liang Z.; Luo J.; Yang Y.; Mu Z.; Zhang X.; Dong H.; Wu F. Ratiometric Optical Thermometer with High Sensitivity Based on Dual Far-Red Emission of Cr^3+^ in Sr_2_MgAl_22_O_36_. Ceram. Int. 2020, 46, 5008–5014. 10.1016/j.ceramint.2019.10.241.

[ref47] Mullins A. L.; Ćirić A.; Zeković I.; Williams J. A. G.; Dramićanin M. D.; Evans I. R. Dual-Emission Luminescence Thermometry Using LaGaO_3_:Cr^3+^, Nd^3+^ Phosphors. J. Mater. Chem. C 2022, 10, 10396–10403. 10.1039/d2tc02011d.

[ref48] Li Z.; Xu S. C.; Zhang C.; Liu X. Y.; Gao S. S.; Hu L. T.; Guo J.; Ma Y.; Jiang S. Z.; Si H. P. High-Performance SERS Substrate Based on Hybrid Structure of Graphene Oxide/AgNPs/Cu Film@pyramid Si. Sci. Rep. 2016, 6, 1 10.1038/srep38539. 27924863 PMC5141445

[ref49] Liu T.; Kim D.; Han H.; bin Mohd Yusoff A. R.; Jang J. Fine-Tuning Optical and Electronic Properties of Graphene Oxide for Highly Efficient Perovskite Solar Cells. Nanoscale 2015, 7, 10708–10718. 10.1039/c5nr01433f. 26030146

[ref50] Lipiäinen T.; Fraser-Miller S. J.; Gordon K. C.; Strachan C. J. Direct Comparison of Low- and Mid-Frequency Raman Spectroscopy for Quantitative Solid-State Pharmaceutical Analysis. J. Pharm. Biomed. Anal. 2018, 149, 343–350. 10.1016/j.jpba.2017.11.013. 29136591

[ref51] Stręk W.; Dereń P.; Jeżowska-Trzebiatowska B. Optical Properties of Cr^3+^ in MgAl_2_O_4_. Phys. B 1988, 152, 3790384 10.1016/0921-4526(88)90006-3.

[ref52] López R.; Gómez R. Band-Gap Energy Estimation from Diffuse Reflectance Measurements on Sol-Gel and Commercial TiO_2_: A Comparative Study. J. Sol-Gel Sci. Technol. 2012, 61, 1–7. 10.1007/s10971-011-2582-9.

[ref53] Marciniak L.; Kniec K.; Elżbieciak-Piecka K.; Trejgis K.; Stefańska J.; Dramićanin M. Luminescence Thermometry with Transition Metal Ions. A Review. Coord. Chem. Rev. 2022, 469, 214671 10.1016/j.ccr.2022.214671.

[ref54] Adachi S. Review - Photoluminescence Properties of Cr^3+^-Activated Fluoride Phosphors. ECS J. Solid State Sci. Technol. 2021, 10, 036001 10.1149/2162-8777/abdfb7.

[ref55] Marciniak L.; Szalkowski M.; Bednarkiewicz A.; Elzbieciak-Piecka K. A Cr^3+^ Luminescence Based Ratiometric Optical Laser Power Meter. J. Mater. Chem. C 2022, 10, 11040–11047. 10.1039/d2tc02348b.

[ref56] Adachi S. Luminescence Spectroscopy of Cr^3+^ in an Oxide: A Strong or Weak Crystal-Field Phosphor?. J. Lumin. 2021, 234, 117965 10.1016/j.jlumin.2021.117965.

[ref57] Elżbieciak-Piecka K.; Drabik J.; Jaque D.; Marciniak L. Cr^3+^-based Nanocrystalline Luminescent Thermometers Operating in a Temporal Domain. Phys. Chem. Chem. Phys. 2020, 22, 25949–25962. 10.1039/d0cp03453c. 33165480

[ref58] Marciniak L.; Bednarkiewicz A.; Strek W. The Impact of Nanocrystals Size on Luminescent Properties and Thermometry Capabilities of Cr, Nd Doped Nanophosphors. Sens. Actuators, B 2017, 238, 381–386. 10.1016/j.snb.2016.07.080.

[ref59] Mączka M.; Gągor A.; Zareba J. K.; Stefańska D.; Drozd M.; Balciunas S.; Šimenas M.; Banys J.; Sieradzki A. Three-Dimensional Perovskite Methylhydrazinium Lead Chloride with Two Polar Phases and Unusual Second-Harmonic Generation Bistability above Room Temperature. Chem. Mater. 2020, 32, 4072–4082. 10.1021/acs.chemmater.0c00973.

[ref60] Hsu H. P.; Li L. C.; Shellaiah M.; Sun K. W. Structural, Photophysical, and Electronic Properties of CH_3_NH_3_PbCl_3_ Single Crystals. Sci. Rep. 2019, 9, 13311 10.1038/s41598-019-49926-z. 31527642 PMC6746810

[ref61] Otto S.; Scholz N.; Behnke T.; Resch-Genger U.; Heinze K. Thermo-Chromium: A Contactless Optical Molecular Thermometer. Chem. – Eur. J. 2017, 23, 12131–12135. 10.1002/chem.201701726. 28430380

[ref62] Gautier R.; Paris M.; Massuyeau F. Exciton Self-Trapping in Hybrid Lead Halides: Role of Halogen. J. Am. Chem. Soc. 2019, 141, 12619–12623. 10.1021/jacs.9b04262. 31339315

[ref63] Kumar V.; Luo Z. A Review on X-ray Excited Emission Decay Dynamics in Inorganic Scintillator Materials. Photonics 2021, 8, 1–27. 10.3390/photonics8030071.

[ref64] Smith M. D.; Jaffe A.; Dohner E. R.; Lindenberg A. M.; Karunadasa H. I. Structural Origins of Broadband Emission from Layered Pb-Br Hybrid Perovskites. Chem. Sci. 2017, 8, 4497–4504. 10.1039/c7sc01590a. 28970879 PMC5618335

[ref65] Bondzior B.; Stefańska D.; Vũ T. H. Q.; Miniajluk-Gaweł N.; Dereń P. J. Red Luminescence with Controlled Rise Time in La_2_MgTiO_6_: Eu^3+^. J. Alloys Compd. 2021, 852, 157074 10.1016/j.jallcom.2020.157074.

[ref66] Kniec K.; Ledwa K.; Marciniak L. Enhancing the Relative Sensitivity of V^5+^, V^4+^ and V^3+^ Based Luminescent Thermometer by the Optimization of the Stoichiometry of Y_3_Al_5-x_Ga_x_O_12_ Nanocrystals. Nanomaterials 2019, 9, 1375 10.3390/nano9101375. 31557921 PMC6836024

[ref67] Stefańska D.; Stefanski M.; Dereń P. J. Unusual Emission Generated from Ca2Mg0.5AlSi1.5O7:Eu2+ and Its Potential for UV-LEDs and Non-Contact Optical Thermometry. J. Alloys Compd. 2021, 863, 1375 10.1016/j.jallcom.2021.158770.

[ref68] Li L.; Tian G.; Deng Y.; Wang Y.; Cao Z.; Ling F.; Li Y.; Jiang S.; Xiang G.; Zhou X. Constructing Ultra-Sensitive Dual-Mode Optical Thermometers: Utilizing FIR of Mn^4+^/Eu^3+^ and Lifetime of Mn^4+^ Based on Double Perovskite Tellurite Phosphor. Opt. Express 2020, 28, 33747 10.1364/oe.409242. 33115034

[ref69] Wang Q.; Liao M.; Mu Z.; Zhang X.; Dong H.; Liang Z.; Luo J.; Yang Y.; Wu F. Ratiometric Optical Thermometer with High Sensitivity Based on Site-Selective Occupancy of Mn^2+^ Ions in Li_5_Zn_8_Al_5_Ge_9_O_36_ under Controllable Synthesis Atmosphere. J. Phys. Chem. C 2020, 124, 886–895. 10.1021/acs.jpcc.9b09379.

[ref70] Brites C. D. S.; Fiaczyk K.; Ramalho J. F. C. B.; Sójka M.; Carlos L. D.; Zych E. Widening the Temperature Range of Luminescent Thermometers through the Intra- and Interconfigurational Transitions of Pr^3+^. Adv. Opt. Mater. 2018, 6, 1701318 10.1002/adom.201701318.

[ref71] Rao X.; Song T.; Gao J.; Cui Y.; Yang Y.; Wu C.; Chen B.; Qian G. A Highly Sensitive Mixed Lanthanide Metal-Organic Framework Self-Calibrated Luminescent Thermometer. J. Am. Chem. Soc. 2013, 135, 15559–15564. 10.1021/ja407219k. 24063306

[ref72] Ananias D.; Brites C. D. S.; Carlos L. D.; Rocha J. Cryogenic Nanothermometer Based on the MIL-103(Tb,Eu) Metal-Organic Framework. Eur. J. Inorg. Chem. 2016, 2016, 1967–1971. 10.1002/ejic.201501195.

[ref73] Cui Y.; Zou W.; Song R.; Yu J.; Zhang W.; Yang Y.; Qian G. A Ratiometric and Colorimetric Luminescent Thermometer over a Wide Temperature Range Based on a Lanthanide Coordination Polymer. Chem. Commun. 2014, 50, 719–721. 10.1039/c3cc47225f. 24287968

[ref74] Wan Y.; Yu L.; Xia T. A Dye-Loaded Nonlinear Metal-Organic Framework as Self-Calibrated Optical Thermometer. Dyes Pigm. 2022, 202, 110234 10.1016/j.dyepig.2022.110234.

