# Supplementary material for: Metal–Organic Framework Optical Thermometer Based on Cr3+ Ion Luminescence
Source: ACS Appl Mater Interfaces. 2023 Jan 30;15(5):7074–82. doi: 10.1021/acsami.2c19957 (PMC9923675; doi:10.1021/acsami.2c19957)
Supplement: Supplementary file 1 — am2c19957_si_001.pdf [file am2c19957_si_001.pdf]

**Supporting information**

Metal-organic framework optical thermometer based on $Cr^{3+}$ ions luminescence

Adam Kabański[a,*], Maciej Ptak[a], Dagmara Stefańska[a*],

[a]Institute of Low Temperature and Structure Research, Polish Academy of Sciences, Box 1410, 50-950 Wrocław 2, Poland

[*]E-mail: a.kabanski@intibs.pl, d.stefanska@intibs.pl

**Table S1.** Quantities of precursors used for the synthesis of the series of $[EA]_2NaCr_xAl_{1-x}(HCOO)_6$

| Real x | Nominal x | Amount of the substrate [g] | | | | | |
|---|---|---|---|---|---|---|---|
| | | $CrCl_3 \cdot 6H_2O$ | $Al(ClO_4)_3 \cdot 9H_2O$ | EA·HCl | HCOONa | *N*-ethylformamide | HCOOH |
| 1.00 | 1.00 | 1.0658 | - | 0.3264 | 0.5985 | 25 ml | 5 ml |
| 0.78 | 0.80 | 0.8530 | 0.3905 | 0.3267 | 0.5985 | | |
| 0.57 | 0.60 | 0.6396 | 0.7803 | 0.3266 | 0.5987 | | |
| 0.30 | 0.40 | 0.3739 | 1.1710 | 0.3265 | 0.5992 | | |
| 0.21 | 0.20 | 0.2139 | 1.5604 | 0.3267 | 0.5989 | | |
| 0.00 | 0.00 | - | 1.9502 | 0.3264 | 0.5987 | | |

**Table S2.** The parameters of the unit cells of the series of $[EA]_2NaCr_xAl_{1-x}(HCOO)_6$

| Parameter | x | | | | | |
|---|---|---|---|---|---|---|
| | 0 | 0.21 | 0.30 | 0.57 | 0.78 | 1 |
| *a* (Å) | 8.038 | 8.051 | 8.065 | 8.079 | 8.092 | 8.102 |
| *b* (Å) | 9.263 | 9.260 | 9.260 | 9.262 | 9.265 | 9.268 |
| *c* (Å) | 14.311 | 14.321 | 14.338 | 14.346 | 14.359 | 14.368 |
| *β* (°) | 123.226 | 123.204 | 123.194 | 123.152 | 123.113 | 123.100 |
| *V* (Å$^3$) | 1065.564 | 1067.598 | 1070.759 | 1073.424 | 1076.531 | 1078.881 |

**Table S3.** The collation of crystal field parameters and energies of electron transitions of the investigated series of $[EA]_2NaCr_xAl_{1-x}(HCOO)_6$

| Parameter | x | | | | |
|---|---|---|---|---|---|
| | 0.21 | 0.30 | 0.57 | 0.78 | 1 |
| $^4A_{2g}\rightarrow{}^2E$ (cm$^{-1}$) | 14549 | 14550 | 14550 | 14551 | 14555 |
| $^4A_{2g}\rightarrow{}^4T_{2g}$ (cm$^{-1}$) | 16610 | 16453 | 16285 | 16112 | 15715 |
| $^4A_{2g}\rightarrow{}^4T_{1g}$ (cm$^{-1}$) | 22764 | 22581 | 22440 | 22383 | 22329 |
| *D*q (cm$^{-1}$) | 1661 | 1645 | 1628 | 1611 | 1572 |
| *B* (cm$^{-1}$) | 601 | 600 | 605 | 622 | 674 |
| *D*q/B | 2.76 | 2.74 | 2.69 | 2.59 | 2.33 |
| *C* (cm$^{-1}$) | 3341 | 3346 | 3337 | 3302 | 3198 |
| *C*/*B* | 5.56 | 5.58 | 5.52 | 5.31 | 4.74 |

**Table S4.** The collation of lifetime time-parameters for a series of $[EA]_2NaCr_xAl_{1-x}(HCOO)_6$

| Parameter | x | | | | |
|---|---|---|---|---|---|
| | 0.21 | 0.30 | 0.57 | 0.78 | 1 |
| $\tau_1$ [ms] | 1.35 | 0.403 | 0.261 | 0.159 | 0.069 |
| $\tau_2$ [ms] | 2.27 | 0.920 | 0.660 | 0.452 | 0.176 |

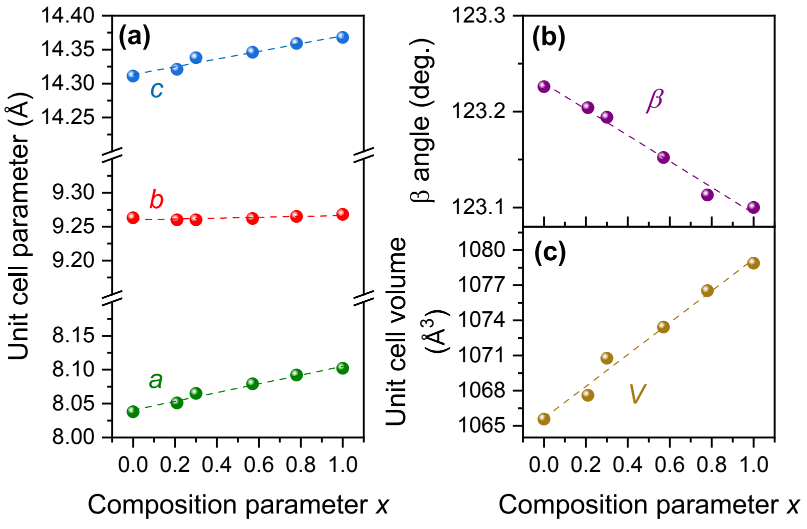


**Figure S1.** Influence of the composition parameter x on the unit cell parameters of the series of $[EA]_2NaCr_xAl_{1-x}(HCOO)_6$: (a) change of *a*, *b*, and *c* parameters; (b) change of the *β* angle; (c) unit cell volumes.

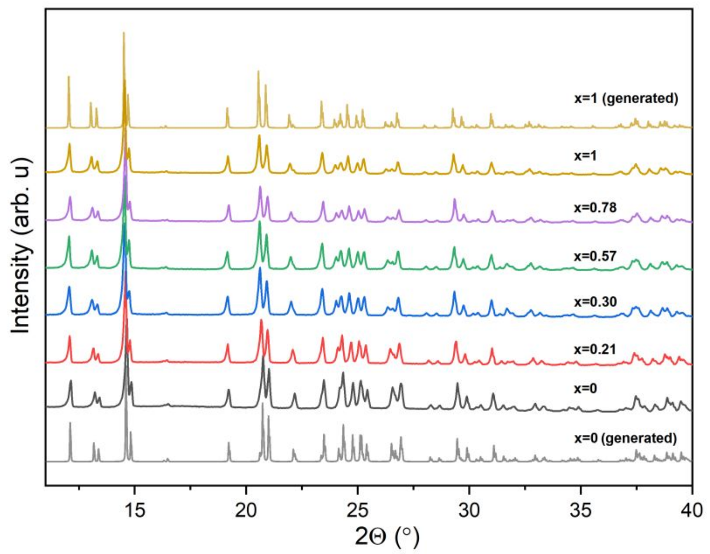


**Figure S2.** XRD patterns for a series of $[EA]_2NaCr_xAl_{1-x}(HCOO)_6$

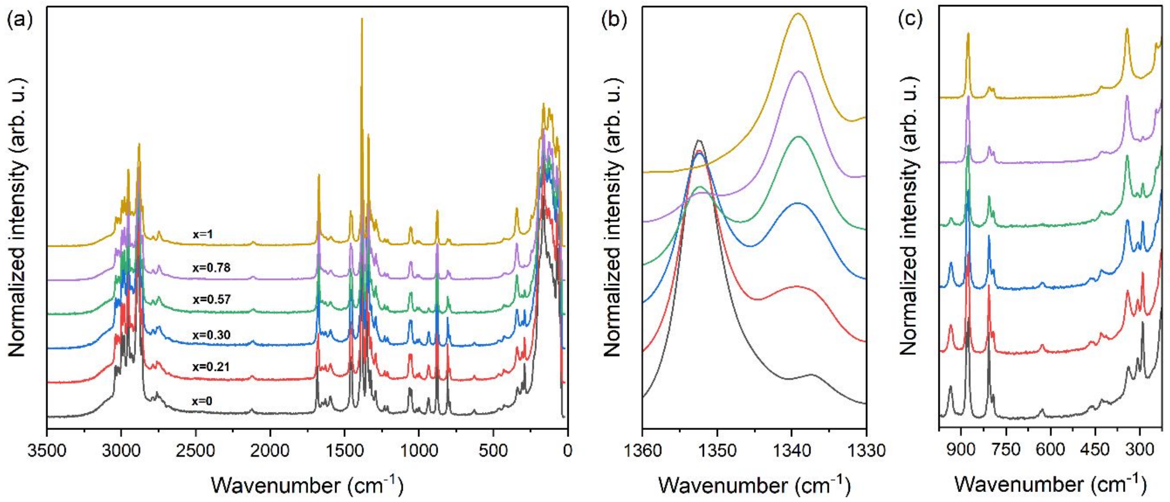


**Figure S3.** (a) Raman spectra for the obtained series of $[EA]_2NaCr_xAl_{1-x}(HCOO)_6$; (b) enlargement of a range within 1330-1360 $cm^{-1}$; (c) enlargement of a range within 225-975 $cm^{-1}$.

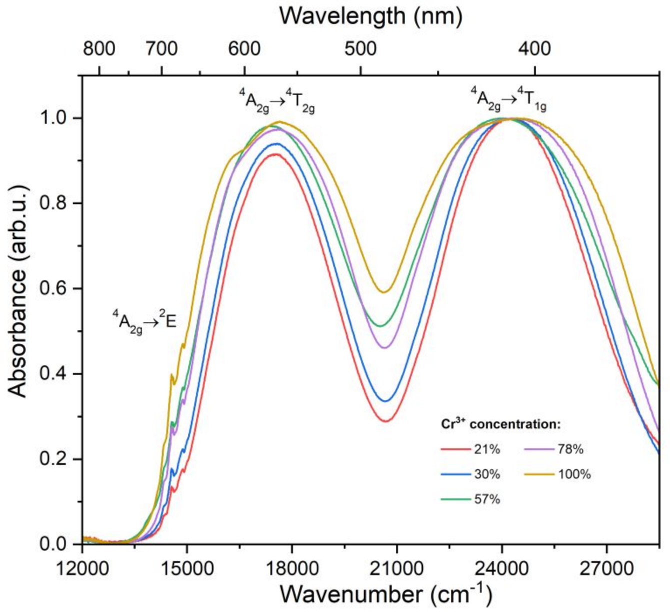


**Figure S4.** Normalized diffuse reflectance spectra of the series of $[EA]_2NaCr_xAl_{1-x}(HCOO)_6$ ( x=1, 0.78, 0.57, 0.30, 0.21, and 0) compounds measured at 300 K

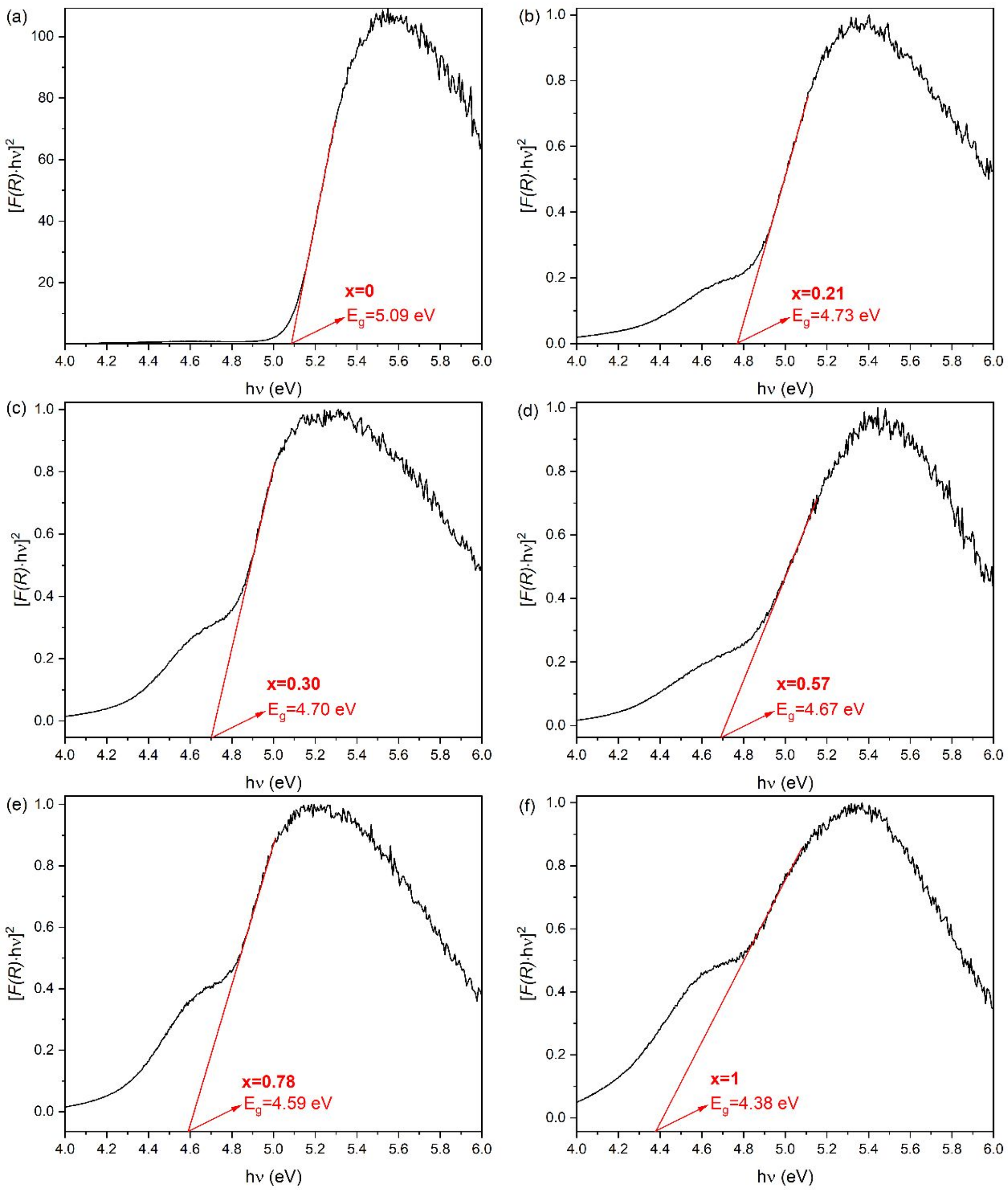


**Figure S5.** (a-f) The energy of band gaps determined with Kubelka-Munk function for the investigated series of $[EA]_2NaCr_xAl_{1-x}(HCOO)_6$. The composition of the individual material is presented in the graph.

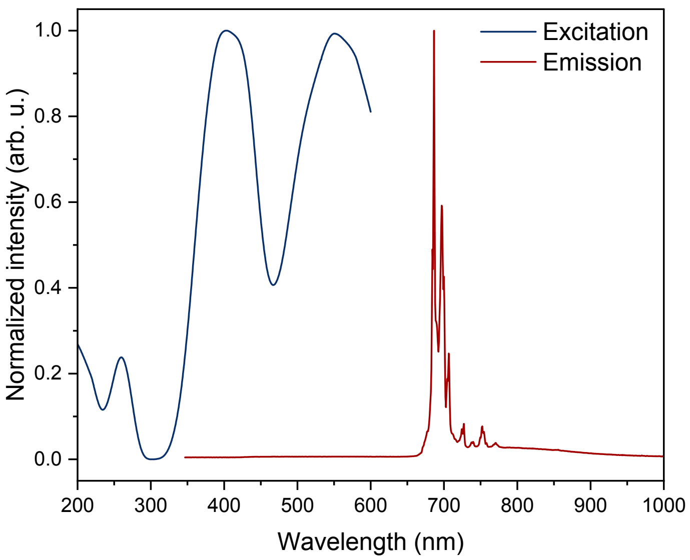


**Figure S6.** The collation of excitation and emission spectra of $[EA]_2NaCr_{0.78}Al_{0.22}(HCOO)_6$ ($\lambda_{exc.}$=405 nm).

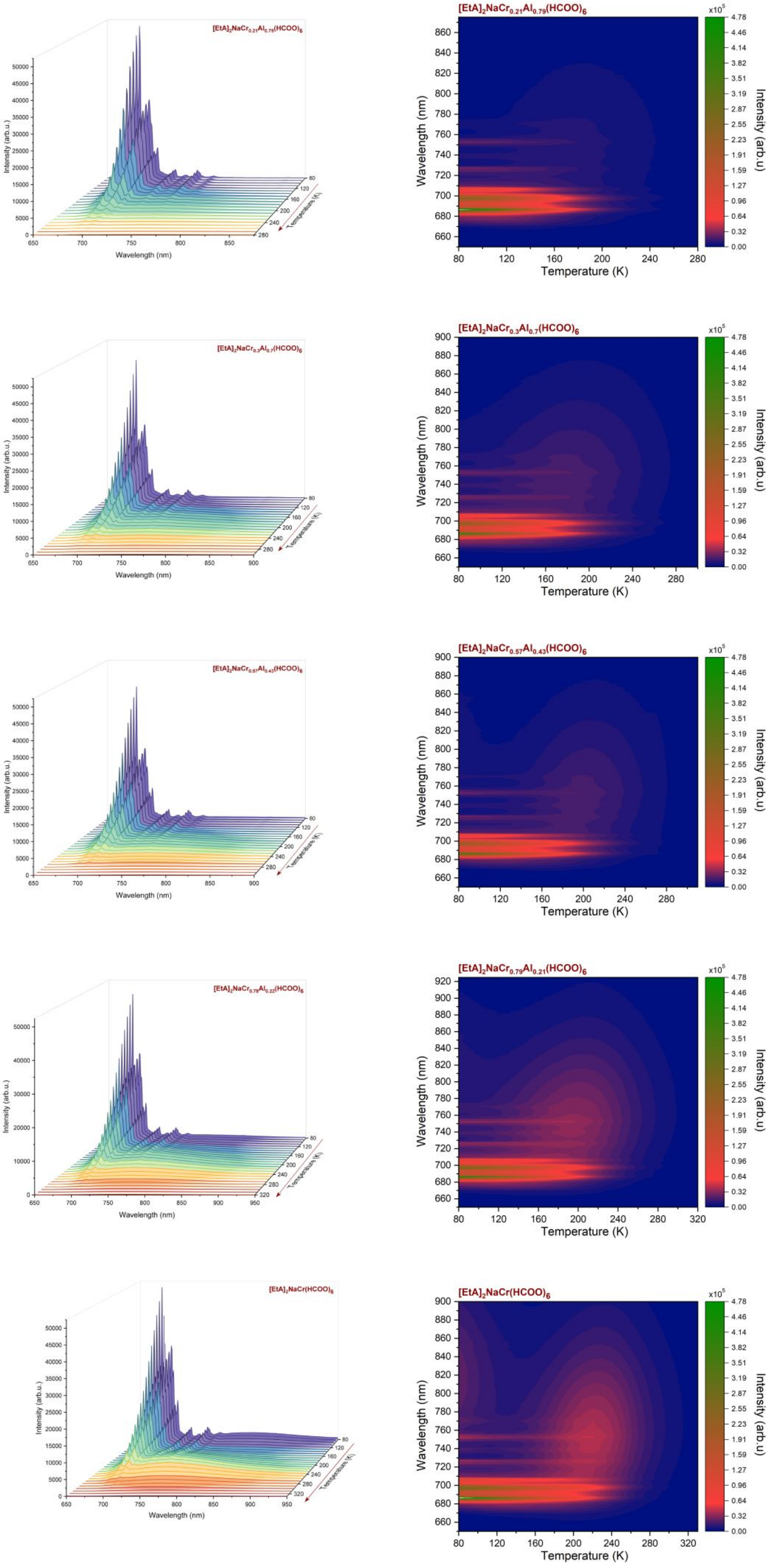
[EtA]$_2$NaCr$_{0.21}$Al$_{0.79}$(HCOO)$_6$
[EtA]$_2$NaCr$_{0.3}$Al$_{0.7}$(HCOO)$_6$
[EtA]$_2$NaCr$_{0.57}$Al$_{0.43}$(HCOO)$_6$
[EtA]$_2$NaCr$_{0.79}$Al$_{0.21}$(HCOO)$_6$
[EtA]$_2$NaCr(HCOO)$_6$
Intensity (arb.u.)
Wavelength (nm)
Temperature (K)
Intensity (arb.u)
x10$^5$

**Figure S7.** The collation of temperature-dependent emission spectra of the investigated materials with evolution map. The composition of the individual material is presented in the graphs.

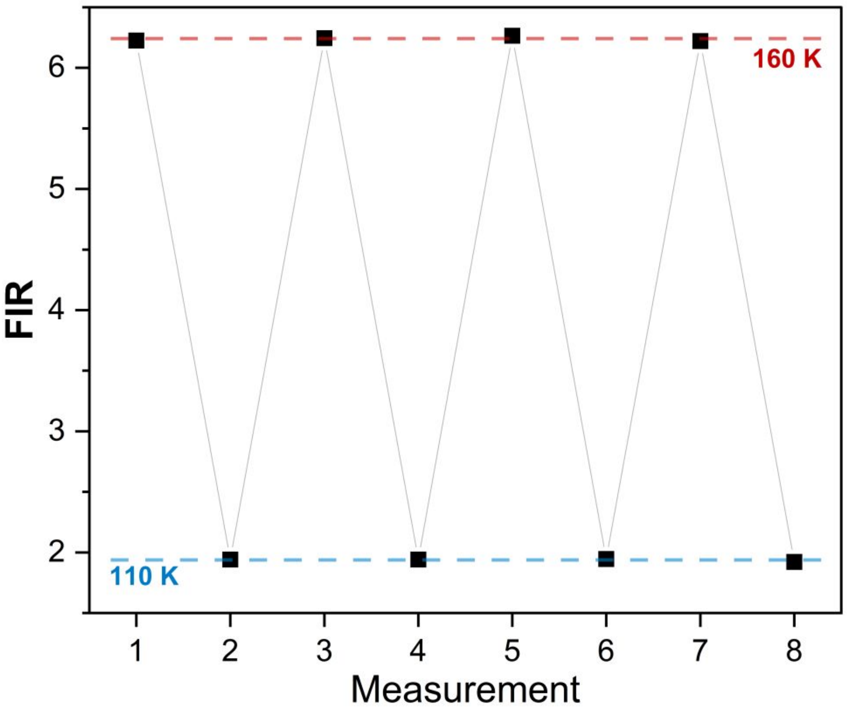


**Figure S8.** Thermometric stability in 110-160 K intervals for $[EA]_2NaCr_{0.21}Al_{0.79}(HCOO)_6$ sample.

**Instruction 1.** – crystal field parameters calculation[1,2]

1. The first step was the DRS spectrum deconvolution within the individual ranges of $^4A_{2g}\rightarrow{}^4T_{2g}$ as well as $^4A_{2g}\rightarrow{}^4T_{1g}$. The obtained values of the less-energetic component of each transition range were presented in Table S3. The energies of $^4A_{2g}\rightarrow{}^2E$ transition for each sample were taken directly from the individual spectra. Having regard to the readability of the instruction, the energy parameters were implemented:
   - *E1* – energy of the $^4A_{2g}\rightarrow{}^2E$ transition;
   - *E2* – energy of the $^4A_{2g}\rightarrow{}^4T_{2g}$ transition;
   - *E3* – energy of the $^4A_{2g}\rightarrow{}^4T_{1g}$ transition.

2. The calculations of the crystal field (Dq) were performed as follows:

$$D\mathrm{q} = \frac{E2_i}{10}, \#(1)$$

   where *i* stands for the individual sample (e.g. $E2_{21\ mol.\%} = 16610\ \mathrm{cm^{-1}}$)

3. The Racah parameters (B and C) were calculated according to the following formulas:

$$x = \frac{E1_i - E2_i}{\mathrm{D}q}, \#(2)$$

$$B = \frac{Dq}{15(x-8)(x^2-10x)}, \#(3)$$

$$C = \frac{E3_i - 7.9B + \frac{1.8B^2}{\mathrm{D}q}}{3.05} \#(4)$$

4. The crystal field strength is described by the radio of crystal field and the Racah parameter – **D*q*/*B*.**

The detailed results of the calculations are presented in Table S3.

[1] B. Henderson and G.F. Imbush, *Optical Spectroscopy of Inorganic Solids*, Clarendon Press, Oxford, 1989, ISBN: 0-19-851372-0

[2] B. Henderson and R.H. Bartram, *Crystal-Field Engineering of Solid-State Laser Materials*, Cambridge: Cambridge University Press, 2000, ISBN 0-521-59349-2
